# IL-3R-alpha blockade inhibits tumor endothelial cell-derived extracellular vesicle (EV)-mediated vessel formation by targeting the β-catenin pathway

**DOI:** 10.1038/s41388-017-0034-x

**Published:** 2017-12-14

**Authors:** Giusy Lombardo, Maddalena Gili, Cristina Grange, Claudia Cavallari, Patrizia Dentelli, Gabriele Togliatto, Daniela Taverna, Giovanni Camussi, Maria Felice Brizzi

**Affiliations:** 1 Department of Medical Sciences, University of Turin, Turin, Italy; 2 Department of Molecular Biotechnology and Health Sciences, Molecular Biotechnology Center (MBC), University of Torino, Torino, Italy

## Abstract

The proangiogenic cytokine Interleukin-3 (IL-3) is released by inflammatory cells in breast and ovarian cancer tissue microenvironments and also acts as an autocrine factor for human breast and kidney tumor-derived endothelial cells (TECs). We have previously shown that IL-3-treated endothelial cells (ECs) release extracellular vesicles (EVs), which serve as a paracrine mechanism for neighboring ECs, by transferring active molecules. The impact of an anti-IL-3R-alpha blocking antibody on the proangiogenic effect of EVs released from TECs (anti-IL-3R-EVs) has therefore been investigated in this study. We have found that anti-IL-3R-EV treatment prevented neovessel formation and, more importantly, also induced the regression of in vivo TEC-derived neovessels. Two miRs that target the canonical wingless (Wnt)/β-catenin pathway, at different levels, were found to be differentially regulated when comparing the miR-cargo of naive TEC-derived EVs (EVs) and anti-IL-3R-EVs. miR-214-3p, which directly targets β-catenin, was found to be upregulated, whereas miR-24-3p, which targets adenomatous polyposis coli (APC) and glycogen synthase kinase-3β (GSK3β), was found to be downregulated. In fact, upon their transfer into the cell, low β-catenin content and high levels of the two members of the “β-catenin destruction complex” were detected. Moreover, c-myc downregulation was found in TECs treated with anti-IL-3R-EVs, pre-miR-214-3p-EVs and antago-miR-24-3p-EVs, which is consistent with network analyses of miR-214-3p and miR-24-3p gene targeting. Finally, in vivo studies have demonstrated the impaired growth of vessels in pre-miR-214-3p-EV- and antago-miR-24-3p-EV-treated animals. These effects became much more evident when combo treatment was applied. The results of the present study identify the canonical Wnt/β-catenin pathway as a relevant mechanism of TEC-derived EV proangiogenic action. Furthermore, we herein provide evidence that IL-3R blockade may yield some significant advantages, than miR targeting, in inhibiting the proangiogenic effects of naive TEC-derived EVs by changing TEC-EV-miR cargo.

## Introduction

Interleukin-3 (IL-3) was originally described as a potent hemopoietic growth factor which acts on progenitor/stem cells and on mature cells [1, 2]. However, the involvement of IL-3 in vascular cell proliferation and activation during physiological and tumor angiogenesis has been extensively documented [3–7]. T lymphocytes and mast cells are the most relevant IL-3-producing cells [8, 9]. Moreover, ovarian and breast cancer-derived tumor infiltrating lymphocytes (CD25/CD4/CD5+TILs) express IL-3 [10]. As originally reported by Deregibus et al. [11], IL-3 also acts as an autocrine factor for tumor-derived endothelial cells (TECs). These data have been further validated in human breast and kidney TECs [12]. Overall, IL-3, present in the tumor microenvironment, can contribute to tumor growth via paracrine and autocrine mechanisms.

The classic paracrine signaling paradigm has been re-evaluated somewhat since it was discovered that both cancer and tumor microenvironment cells generate membrane-enclosed packets, called extracellular vesicles (EVs). EVs from different origin contain both a common set of molecules and components specific of the cell of origin. EVs released from cancer cells contain proteins reflecting their endosomal origin together with cellular oncogenic drivers, phosphorylated proteins and miRNAs [13–15]. EVs have also received increasing levels of attention in recent years because of their role in regulating and transferring active molecules that are responsible for tumor metastasis [16]. Therefore, to inhibiting EV functional effects would most likely yield some significant advantages in the treatment of neoplasm. Unlike soluble factors secreted by cells, EVs bring functional molecules, which serve as intra- and intercellular communicators, locally and systemically [17]. EVs can promote tumor growth and metastasis even by inducing angiogenesis [13–16, 18–20]. This event has been extensively documented in tumor cell-derived EVs [21]. However, endothelial cells (ECs) themselves can release EVs in response to angiogenic stimuli [22] thus also contributing to the angiogenic activity of growing microvessels.

Several studies have described the Wnt-β-catenin pathway as a crucial regulator of EC fate during embryonic development and tumor angiogenesis [23–28]. Unlike in normal mature cells, the abnormal activation of the Wnt/β-catenin signal occurs during cancer development [24, 25, 29–31]. The canonical Wnt/β-catenin signaling pathway initiates by the binding of the Wnt ligand to its receptor, Frizzled (FZD), and the LDL receptor-related proteins 5 or 6 [29, 32–34]. As a consequence, the cytoplasmic protein Disheveled (Dvl) is phosphorylated and the detachment of β-catenin from the “β-Catenin destruction complex”, which consists of a number of members including the adenomatous polyposis coli (APC), Axin the glycogen synthase kinase-3β (GSK3β) and the casein kinase 1α (CK1), is enabled [29,^32^–^34^]. Stabilized β-catenin translocates into the nucleus where it forms a β-catenin-T-cell factor/lymphoid enhancer factor (TCF/LEF) transcriptional complex and induces the transcription of some of its downstream genes, such as c-myc and cyclin D1 [29, 32–34]. In the absence of Wnt, cytoplasmic β-catenin is phosphorylated by activated GSK3β and undergoes proteasomal degradation [29, 32–34]. The role of Wnt/β-catenin in driving carcinogenesis, cancer progression and metastasis has been extensively documented in many tumors [24, 25, 29–31] Furthermore, evidence to support the relevance of the interaction between miRs and the Wnt/β-catenin pathway in cancer has recently emerged [24, 35]. However, the contribution of miRs to regulating the Wnt/β-catenin signaling pathway in tumor angiogenesis has only been poorly investigated.

We have recently provided evidence that EC-derived EVs mediate the transfer of activated proteins and miRs in inflammatory sites containing IL-3, which boosts wound healing [22]. These observations have led us to hypothesize that the release of IL-3 by TECs may also support the establishment of a favorable milieu via EVs. In this study, we have therefore decided to investigate the proangiogenic action of TEC-derived EVs and the impact of an anti-IL-3R-alpha blocking antibody, which is currently under investigation in acute myeloid leukemia patients [36], on the proangiogenic effect of EVs released from TECs.

## Results

### EVs released by TECs subjected to IL-3 receptor blockade reduce TEC proliferation

**Fig. 1:**
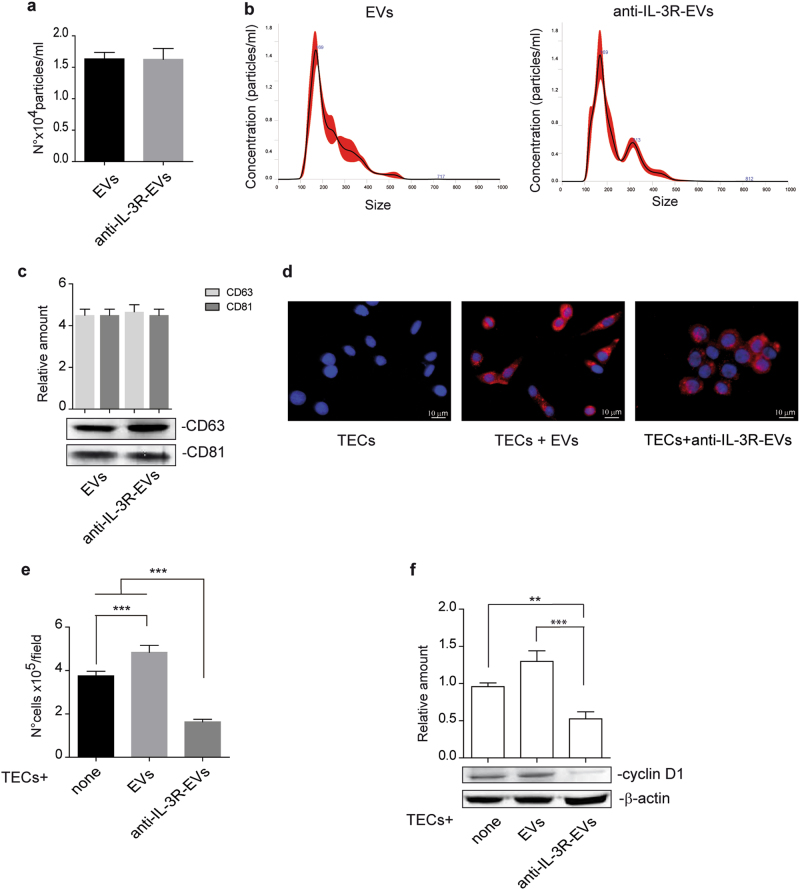
Internalization and proliferation in response to TEC-derived EVs and anti-IL-3R-EVs. **a** Number of EV particles (mean ± SD) was calculated per ml. Data refer to EV-derived from untreated TECs (EVs) or anti-IL-3Rα blocking antibody-treated TECs (anti-IL-3R-EVs) (*n* = 4, unpaired *t*-test). **b** Representative images of NanoSight analyses performed on the 100 k fraction of TEC-derived EVs, showing a mean dimension, for both EVs, of 169 nm (*n* = 3). **c** TEC-derived EVs were analyzed for CD63 and CD81 exosomal markers by western blot analysis (*n* = 4, unpaired *t*-test). **d** Representative confocal microscopy images of TECs, either untreated or treated for 3 h with the indicated PKH26-labeled EVs, to evaluate EV up-take TECs. (*n* = 4). Scale bars indicate 10 μm (40× magnification). **e** Cell proliferation assay was performed in TECs untreated (none) or treated for 24 h with EVs or anti-IL-3R-EVs and reported as number of cells per field (mean ± SD, 20× magnification) (*n* = 5) (***p < 0.001, none vs EVs; ***p < 0.001, none and EVs vs anti-IL-3R-EVs, one-way ANOVA). **f** TECs, either alone or stimulated with EVs or anti-IL-3R-EVs, were lysed and analyzed for cyclin D1 content. Protein levels were normalized to β-actin content (**p < 0.01, none vs anti-IL-3R-EVs; ***p < 0.001, EVs vs anti-IL-3R-EVs, one-way ANOVA) (*n* = 4)

It has been reported that ECs, when subjected to IL-3, release EVs which act as paracrine proangiogenic mediators [22]. To investigate whether and how TEC-derived EVs induce proangiogenic cues and whether this effect may be prevented by interfering with IL-3-mediated signaling, EVs were recovered after treating TECs with an antibody against the IL-3 receptor alpha subunit (anti-IL-3R-EVs). Firstly, we investigated whether differences in EV release occurred. No differences in the number (Fig. 1a) and size (Fig. 1b) of EVs released were found. Exosome expression markers are reported in Fig. 1c. In order to evaluate whether EVs released under both experimental conditions could be internalized to act as paracrine mediators, EVs labeled with PKH26 dye were assayed for TEC internalization. Figure 1d shows that both EVs and anti-IL-3R-EVs can be incorporated by TECs. To exclude the possibility that the anti-IL-3R antibody bound to EVs could mediate the effects of anti-IL-3R-EVs, Western blot analysis was performed on anti-IL-3R EV pellet and anti-IL-3R-EV-depleted conditioned medium (CM). Supplementary Fig. 1a-b clearly demonstrated the presence of the anti-IL-3R antibody in the anti-IL-3R-EV-depleted CM, but not in the anti-IL-3R-EV pellets. EV-depleted CM and EV pellet from untreated TECs served as controls. Functional studies were also performed by exploring the effect that EVs and anti-IL-3R-EVs had on TEC proliferation. Unstimulated TECs were used as an internal control. It was found that EVs promoted TEC proliferation, as shown in Fig. 1e. This effect was significantly reduced in TECs treated with anti-IL-3R-EVs. Low cyclin D1 levels were consistently detected in TECs that had been stimulated with anti-IL-3R-EVs (Fig. 1f). No differences in the number of apoptotic cells were found (data not shown).

### Anti-IL-3R-EVs prevent and impair the growth of tumor vessels

**Fig. 2:**
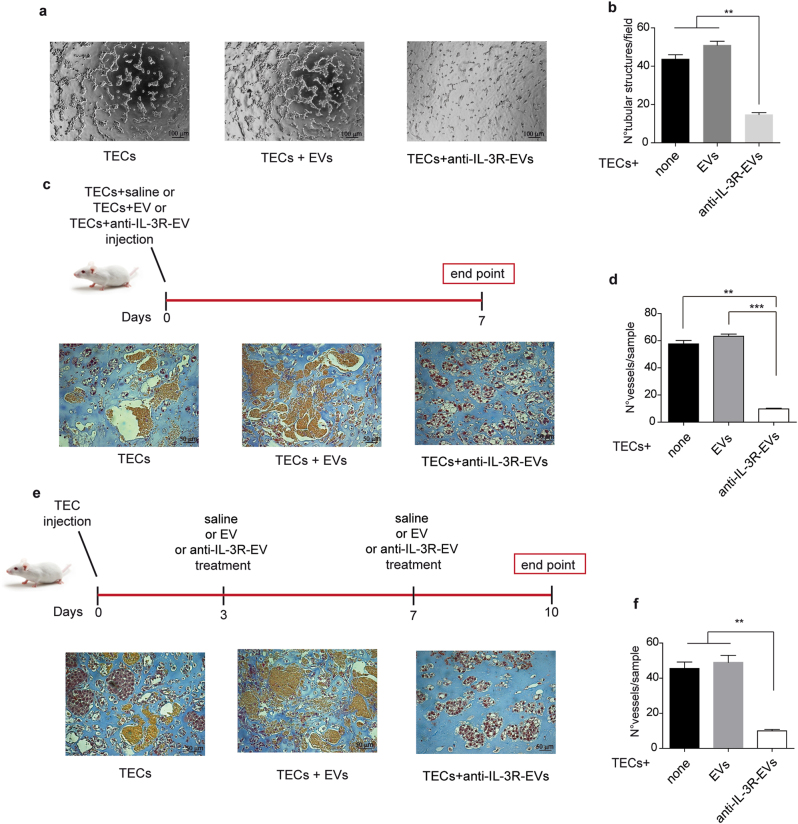
Anti-IL-3R-EVs impair vessel formation. **a**, **b** Representative photomicrographs of an in vitro angiogenesis assay, showing tube-like structure formation by TECs, either untreated or treated with 7 × 10^3^/cell EVs or anti-IL-3R-EVs. Data are reported in the histogram as number ± SD of tubular structures per field (**p < 0.01, none and EVs vs anti-IL-3R-EVs, one-way ANOVA) (*n* = 4, 10× magnification). Scale bars indicate 100 μm. **c**, **d** Representative images of an in vivo angiogenesis assay, performed by injecting Matrigel matrices containing either 1 × 10^6^ TECs and 1 × 10^5^/cell of EVs or anti-IL-3R-EVs into the flank of SCID mice. Saline was the internal control. As shown by the timeline, plugs were recovered on day 7 after implantation. Data are reported in the histogram as number ± SD of vessels per sample (**p < 0.01, none vs anti-IL-3R-EVs; ***p < 0.001, EVs vs anti-IL-3R-EVs, one-way ANOVA) (20× magnification). Scale bars indicate 50 μm. **e**, **f** Representative images of an in vivo angiogenesis assay to evaluate the ability of anti-IL-3R-EVs to impair the growth of tumor vessels. The timeline and the endpoint are indicated. Data are reported in the histogram as number ± SD of vessels per sample (**p < 0.01, none and EVs vs anti-IL-3R-EVs, one-way ANOVA) (20× magnification). Scale bars indicate 50 μm

An in vitro angiogenic assay was performed in order to evaluate whether anti-IL-3R-EVs interfere with TECs’ ability to form neovessels. The treatment of TECs with anti-IL-3R-EVs was associated with reduced formation of tube-like structures, as shown in Fig. 2a, b. Two different in vivo approaches were used to confirm this effect. TECs, pre-incubated with EVs and anti-IL-3R-EVs, were included in Matrigel and injected subcutaneously into severe combined immunodeficient (SCID) mice. 7 days after implantation, plugs were recovered and analyzed for the presence of vessels. As shown in Fig. 2c, d, anti-IL-3R-EVs almost completely inhibited the angiogenetic capability of TECs. In order to evaluate whether anti-IL-3R-EVs could also be exploited as a therapeutic option, in vivo experiments were performed by injecting Matrigel-containing TECs into SCID mice. Either EVs, anti-IL-3R-EVs or saline were administered locally on days 3 and 7 after implantation. The plugs were recovered and analyzed on day 10. It was found that mice treated with anti-IL-3R-EVs failed to form organized vessels in vivo, unlike saline- or EV-treated animals, as shown in Fig. 2e, f. These results indicate that anti-IL-3R-EVs are active in preventing and impairing the growth of tumor neovessels.

### Anti-IL-3R-EV-mediated biological effects depend on their miR cargo variation

**Fig. 3:**
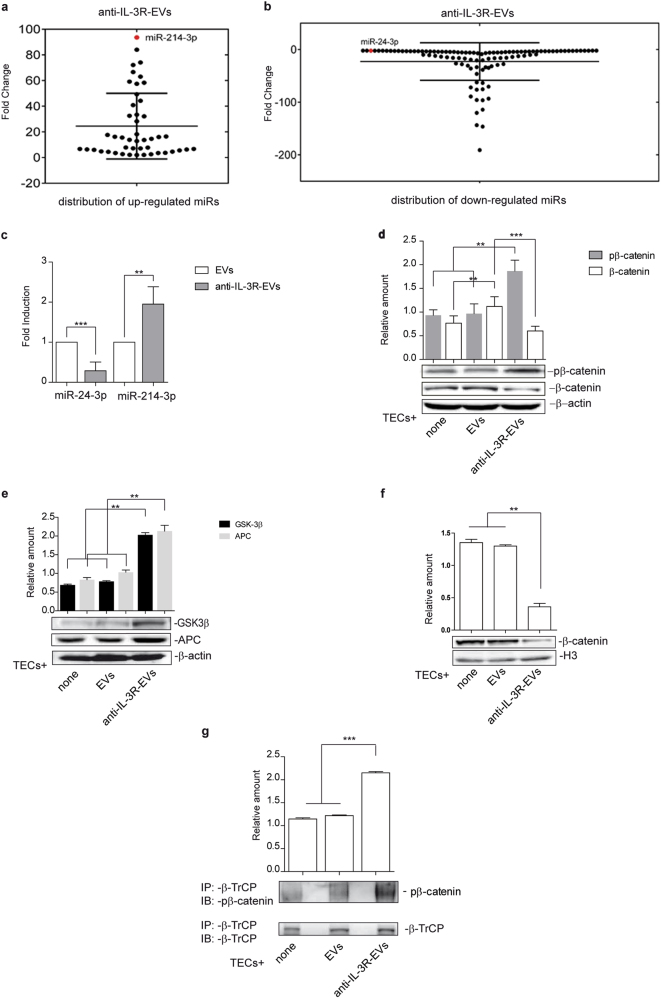
Anti-IL-3R-EV miR distribution and modulation of the β-catenin signaling pathway upon EV and anti-IL-3R-EV treatment **a** Dot plot graph distribution of the 47 significant upregulated miRs in anti-IL-3R-EVs, compared to EVs. Each dot represents the fold change value of each miR. Only miRs with fold change ≥ 2 are reported. miR-214-3p (red dot) was found as the most upregulated miR in anti-IL-3R-EVs (fold change: 93.63 ± 4.52). **b** Dot plot graph distribution of the 107 significant downregulated miRs in anti-IL-3R-EVs, compared to EVs. miRs with fold change ≤ 2 are reported. miR-24-3p (red dot) was found to be downregulated (fold change: -2.17 ± 0.08). **c** miR‐24-3p and miR-214-3p expression was validated by qRT–PCR on EVs and anti-IL-3R-EVs and data normalized to RNU6B (*n* = 4) (**p < 0.01, EVs vs anti-IL-3R-EVs for miR‐24-3p and miR-214-3p, unpaired *t*-test). **d** Total cell extracts from TECs, treated as indicated, were subjected to Western blot analysis to evaluate pβ-catenin and β-catenin content, normalized to β-actin (*n* = 4) (**p < 0.01 none and EVs vs anti-IL-3R-EVs for pβ-catenin; **p < 0.01 none vs EVs for β-catenin; ***p < 0.001 EVs vs anti-IL-3R-EVs for β-catenin, one-way ANOVA). **e** Cytoplasmic extracts from TECs, treated as indicated, were subjected to Western blot analysis to evaluate APC and GSK 3β content, normalized to β-actin (*n* = 4) (**p < 0.01, none and EVs vs anti-IL-3R-EVs, one-way ANOVA). **f** Nuclear extracts from TECs, treated as above, were analyzed for β-catenin content and normalized to H3 (*n* = 3) (**p < 0.01, none and EVs vs anti-IL-3R-EVs, one-way ANOVA). **g** Cytoplasmic extracts from untreated or treated TECs, as indicated, were immunoprecipitated with anti-β-TrCP antibody, subjected to SDS–PAGE and immunoblotted with anti-β-catenin and anti-β-TrCP antibodies (***p < 0.001, none and EVs vs anti-IL-3R-EVs, one-way ANOVA)

EV biological activity relies on the transfer into recipient cells of proteins, lipids and genetic material, including miRs, [13, 14, 17, 37–39]. Anti-IL-3R-EV miR content was therefore compared to that of EVs in order to investigate the molecular mechanisms that account for in vivo results. A complete panel of mature miRs was screened. Figure 3a, b shows the distribution of miRs that were upregulated and downregulated in anti-IL-3R-EVs. miR fold change is reported in Supplementary Tables 1 and 2. Software prediction analyses on modulated miRs in anti-IL-3R-EV cargo identified two miRs that were differentially modulated and that shared a common pathway; the canonical Wnt-β-catenin pathway. These miRs were found to act on different levels; miR-214-3p, which is upregulated, directly targets β-catenin [40], miR-24-3p, which is downregulated, targets two different “β-catenin destruction complex” genes, APC and GSK3β (TarBase v7.0) [41]. The expression of miR-24-3p and miR-214-3p in EVs and anti-IL-3R-EVs was validated by real-time PCR (Fig. 3c). It was then decided to analyze the expression of phosphorylated and unphosphorylated β-catenin in total cell lysates in TECs treated with EVs and anti-IL-3R-EVs in order to investigate the possibility that miR-24-3p and miR-214-3p, carried by anti-IL-3R-EVs, may interfere with the β-catenin signaling pathway. The results reported in Fig. 3d clearly only demonstrate an increase in phosphorylated, and a decrease in unphosphorylated β-catenin content in TECs subjected to anti-IL-3R-EV treatment. Supplementary Fig. 2a, b show that, in TECs, the level of phosphorylated and unphosphorylated β-catenin did not change upon anti-IL-3R treatment.

### The canonical Wnt/β-Catenin pathway is targeted by anti-IL-3R-EVs

**Fig. 4:**
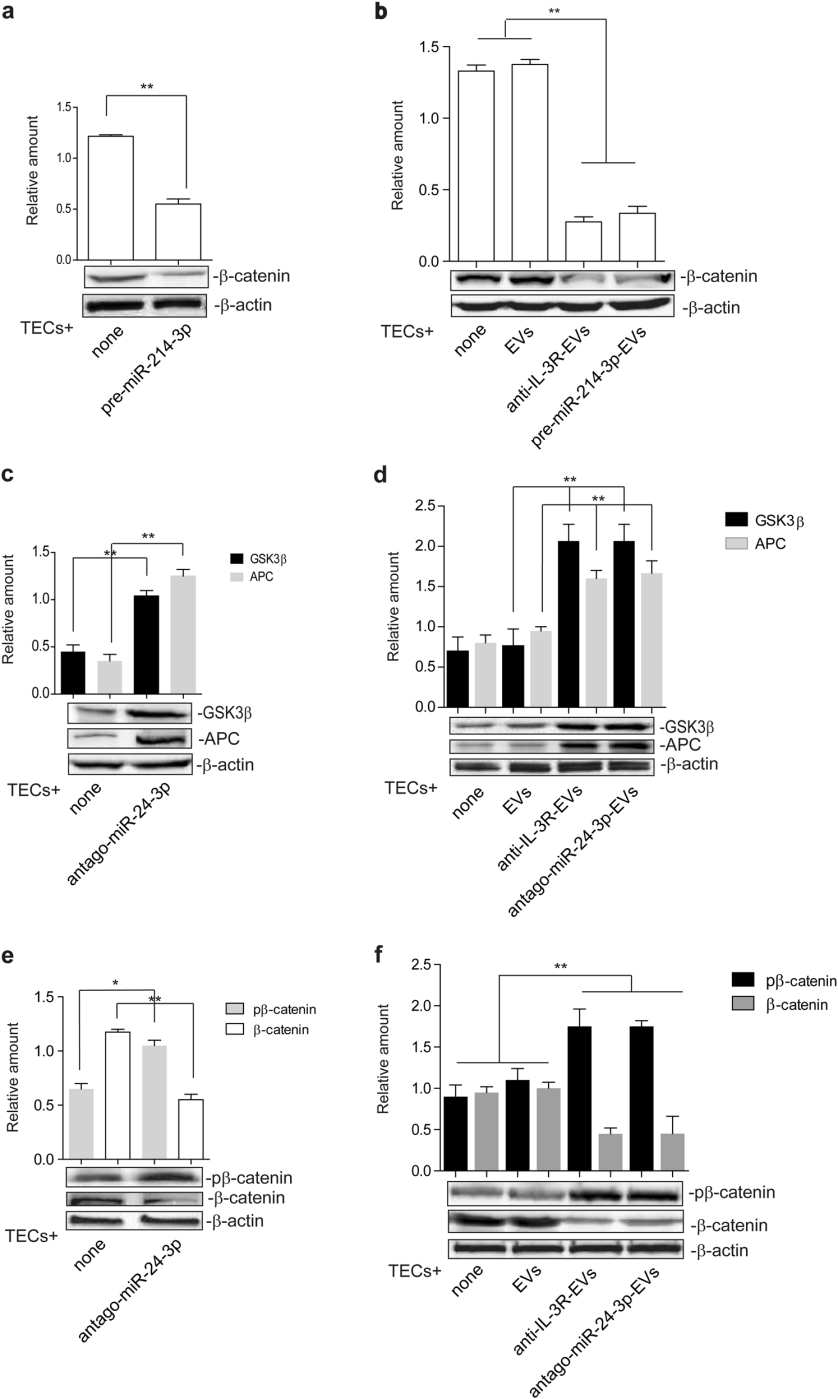
EV miR content controls β-catenin activation. **a** TECs that over-express miR-214-3p were subjected to SDS–PAGE to evaluate β-catenin content, normalized to β-actin. Untransfected TECs were used as internal controls (none) (*n* = 5) (**p < 0.01, none vs pre-miR-214-3p, unpaired *t*-test). **b** Cell extracts from TECs, left untreated or treated with EVs, anti-IL-3R-EVs or EVs enriched in miR-214-3p, were analyzed for β-catenin content, normalized to β-actin (*n* = 5) (**p < 0.01, none and EVs vs anti-IL-3R-EVs and pre-miR-214-3p-EVs, one-way ANOVA). **c**, **d** Cytoplasmic extracts from TECs depleted of miR-24-3p (antago-miR-24-3p) (**c**) and from TECs treated with antago-miR-24-3p-EVs (**d**) were analyzed by Western blot for APC and GSK3β content (**p < 0.01, none vs antago-miR-24-3p in (c), unpaired *t*-test, and **p < 0.01, EVs vs anti-IL-3R-EVs and antago-miR-24-3p-EVs in **d**, one-way ANOVA). **e**, **f** Cell extracts from antago-miR-24-3p TECs (**e**) and from TECs, treated as above (**f**), were analyzed for pβ-catenin and β-catenin content, normalized to β-actin (*n* = 4) (*p < 0.05, for pβ-catenin and **p < 0.01, for β-catenin, none vs antago-miR-24-3p in **e**, unpaired *t*-test, and **p < 0.01, none and EVs vs anti-IL-3R-EVs and antago-miR-24-3p-EVs in **f**, one-way ANOVA)

**Fig. 5:**
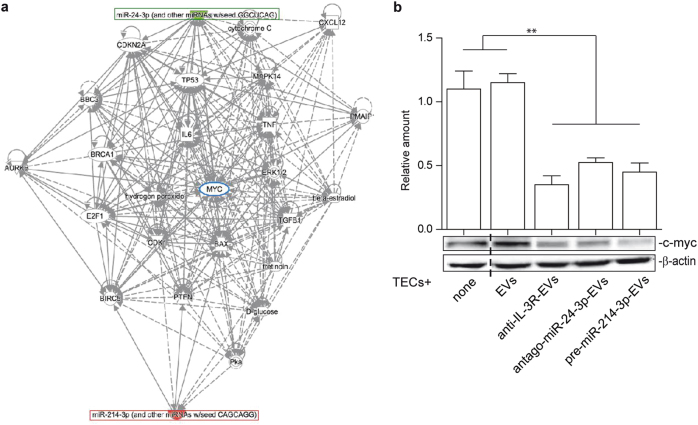
miR-24-3p and miR-214-3p integrated interaction-networks merge in c-myc **a** Network analysis between miR-24-3p/miR-214-3p and mRNA targets. Lines represent interactions between genes and miRs predicted by the IPA Software: indirect interactions (dotted lines), direct interactions (continuous lines). **b** TECs treated as indicated were lysed and analyzed for c-myc content, normalized to β-actin content (*n* = 5) (**p < 0.01, none and EVs vs anti-IL-3R-EVs, antago-miR-24-3p-EVs and pre-miR-214-3p-EVs, one-way ANOVA)

APC and GSK3β, known miR-24-3p targets, were evaluated to gain further insight into the anti-IL-3R-EV mechanism of action. As shown in Fig. 3e, both APC and GSK3β levels increased after anti-IL-3R-EV treatment. Conversely, their expression did not change in anti-IL-3R-treated TECs (Supplementary Fig. 2b). APC and GSK3β were neither detected in naive nor in anti-IL-3R-EVs (Supplementary Fig. 2b). β-catenin was strongly reduced in the nuclear fraction (Fig. 3f), which is consistent with the increase in “β-catenin destruction complex” components. This was further validated by immunoprecipitation experiments using an antibody against the beta-transducing repeats-containing proteins (β-TrCP), which act as the substrate recognition subunits for the SCF (Skp1–Cullin1–Fbox protein)^β-TrCP^ E3 ubiquitin ligases [42]. Indeed, a complex containing the phosphorylated β-catenin and β-TrCP was detected upon anti-IL-3R-EV treatment (Fig. 3g). Since β-catenin content was reduced in the total lysates of anti-IL-3R-EV-treated TECs (Fig. 3d), it was decided to determine whether the enrichment of miR-214-3p in anti-IL-3R-EVs could exert a direct β-catenin expression effect. Gain-of-function experiments were therefore performed. TECs were transfected with pre-miR-214-3p and EVs were recovered (pre-miR-214-3p-EVs). Transfection efficacy was confirmed by real-time PCR (rt-PCR) in both cells and EVs (Supplementary Fig. 3a, b). The effects of pre-miR-214-3p on β-catenin expression were initially evaluated in transfected cells (Fig. 4a) and then in TECs treated with pre-miR-214-3p-EVs (Fig. 4b). As expected, β-catenin content was reduced both in transfected cells and in TECs stimulated with pre-miR-214-3p-EVs. The contribution of miR-24-3p to anti-IL-3R-EV-mediated action was then evaluated by performing loss-of-function experiments. TECs were transfected with antago-miR-24-3p and EVs (antago-miR-24-3p-EVs) were recovered. Antago-miR-24-3p depletion was confirmed by rt-PCR in cells and EVs (Supplementary Fig. 1c, d). As shown in Fig. 4c, d, increased APC and GSK3β content was detected in both transfected cells and in antago-miR-24-3p-EV-treated TECs. Furthermore, increased levels of phosphorylated β-catenin and a decrease in unphosphorylated β-catenin were found (Fig. 4e, f). The integrated miR-24-3p and miR-214-3p interaction-network identified c-myc as one of their downstream nodes (Fig. 5a). c-myc expression was therefore analyzed on TECs treated with different EVs. As shown in Fig. 5b, anti-IL-3R-EVs, antago-miR-24-3p- and pre-miR-214-3p-EVs failed to induce c-myc expression, unlike EVs.

### miR-214-3p and miR-24-3p are both involved in anti-IL-3R-EV-antiagiogenic effects

**Fig. 6:**
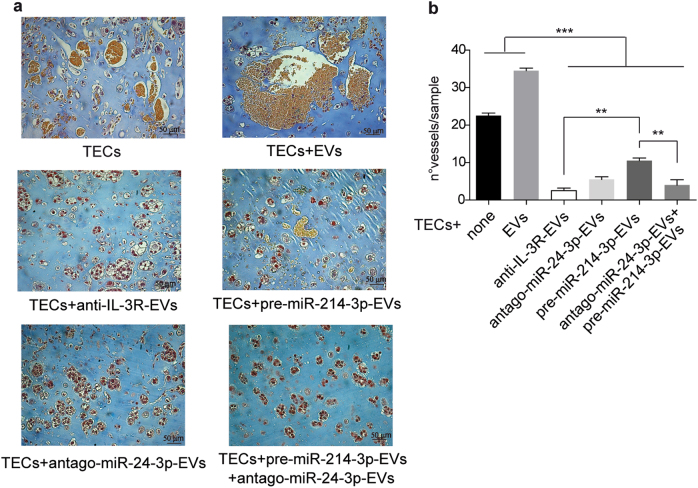
Effects of antago-miR-24-3p-EVs and pre-miR-214-3p-EVs on TEC-derived vessels **a**, **b** Representative images of an in vivo angiogenesis assay of TECs treated with antago-miR-24-3p-EVs and pre-miR-214-3p-EVs either alone or in combination. Untreated TECs and TECs treated with EVs and anti-IL-3R-EVs were also reported. Data are reported in the histogram as number ± SD of vessels per sample (***p < 0.001, none and EVs vs others experimental conditions; **p < 0.01, anti-IL-3R-EVs vs pre-miR-214-3p-EVs and pre-miR-214-3p-EVs vs antago-miR-24-3p-EVs + pre-miR-214-3p-EVs, one-way ANOVA) (20× magnification). Scale bars indicate 50 μm

**Fig. 7:**
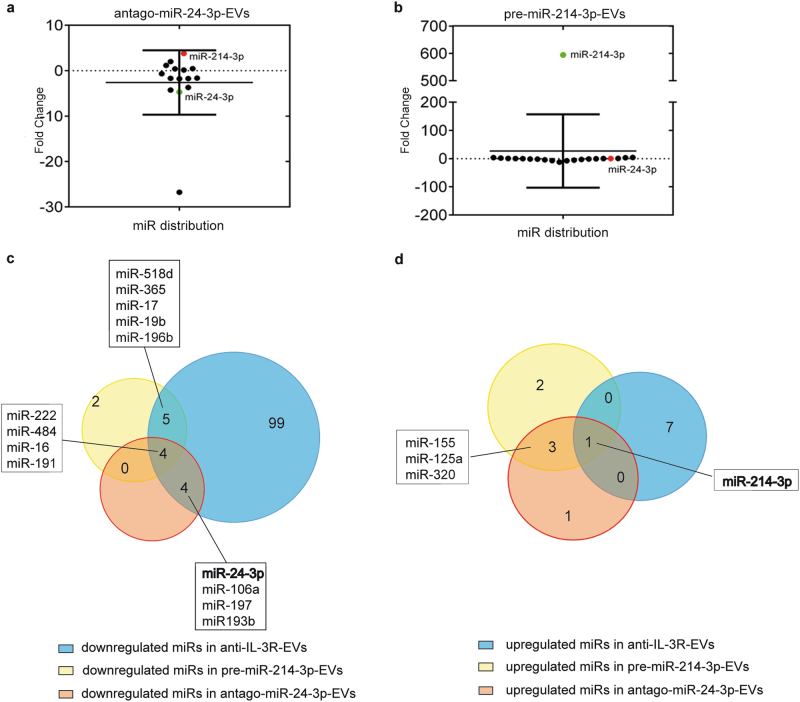
miR distribution in antago-miR-24-3p-EVs and pre-miR-214-3p-EVs. **a** Dot plot distribution graph of the 15 modulated miRs in antago-miR-24-3p-EVs compared to EVs. Dots represent fold change values for each miR. miR-214-3p (red dot) was found to be significantly upregulated in antago-miR-24-3p EVs (foldchange: 3.80 ± 1.00). **b** Dot plot distribution graph of the 21 modulated miRs in pre-miR-214-3p-EVs compared to EVs. miR-24-3p (red dot) was found to be almost unmodulated in pre-miR-214-3p-EVs (foldchange: 0.26 ± 0.14). **c** Venn diagram of downregulated miRs, identified in anti-IL-3R-EVs, antago-miR-24-3p EVs and pre-miR-214-3p EVs, are reported. The diagram shows an overlap of common miRs across different EVs **d** Venn diagram of upregulated miRs identified in anti-IL-3R-EVs, antago-miR-24-3p EVs and pre-miR-214-3p EVs. The diagram shows an overlap of common miRs among different EVs

**Fig. 8:**
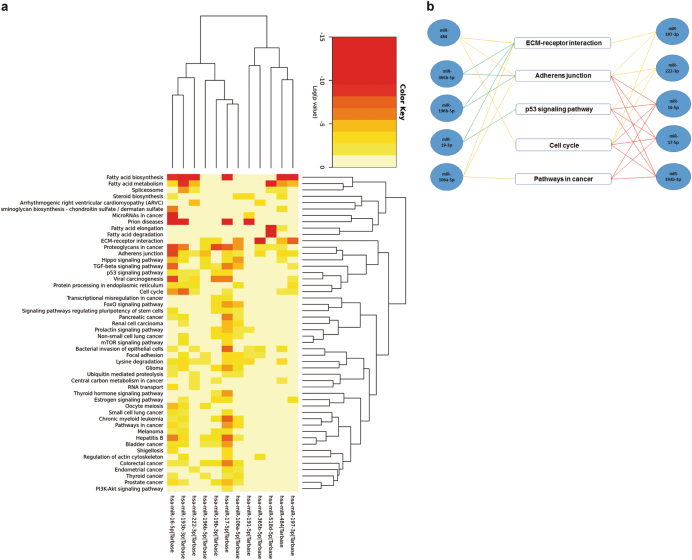
miR/pathway heat map representation and KEGG pathways—miRNA network. **a** Meta-analysis algorithm of the most significant pathways targeted by miR-222-3p, miR-16-5p, miR-484, miR-17-5p, miR-106a-5p, miR-365b-5p, miR-196b-5p, miR-19b-3p, miR-197-3p, miR-193b-3p, miR-518d-5p and miR-191-5p. Relevant correlation with the following pathways was found; cell cycle pathway (*p*-value = 9.55^−11^), adherens junction (*p*-value = 1.0^−325^), pathway in cancer (*p*-value = 1.9^−8^), ECM receptor interaction (*p*-value = 1.0^−325^) and p53 signaling pathway (*p*-value = 1.27^−8^). (Image from DIANA-miRpath software). Color notation identifies Log (*p*-value). **b** Representative scheme of DIANA miRpath analysis between miRs and correlated pathways. miRs were linked with pathways by lines of different colors. Red lines: miRs involved in 4 pathways; yellow lines: miRs involved in 3 pathways; green lines: miRs involved in two pathways

Functional studies were performed in vivo to confirm the above data. Differing EV sources were used to treat mice on day 3 and 7 after TEC injection. As shown in Fig. 6a, b, both pre-miR-214-3p-EVs and antago-miR-24-3p-EVs strongly reduced TEC-derived vessels in vivo. However, it was found that, unlike antago-miR-24-3p-EVs, pre-miR-214-3p-EVs were less effective in reducing vessel growth than anti-IL-3R-EVs, while the combo treatment completely recapitulated the anti-IL-3R-EV anti-angiogenic effect. In order to gain further insight into the mechanisms behind the differences in antago-miR-24-3p-EV and pre-miR-214-3p-EV in vivo effects, miR cargo was analyzed and their fold change is reported in Supplementary Table 3 and 4. Figure 7a, b shows how up-upregulated and downregulated miRs are distributed. It is worth noting that antago-miR-24-3p-EVs were found to be enriched in miR-214-3p (Fig. 7a), while no changes in miR-24-3p content were detected in pre-miR-214-3p-EVs (Fig. 7b). This finding is consistent with the reduced β-catenin expression reported in Fig. 4e, f. A cross-match of miRs, carried by different EVs, was also performed. As shown in the Venn diagram (Fig. 7c), 4 miRs were downregulated in pre-miR214-3p-EVs, antago-miR-24-3p-EVs and in anti-IL-3R-EVs (Fig. 7c). While the Venn diagram in Fig. 7d shows the cross-match of upregulated miRs shared by different EVs, only miR-214-3p was upregulated in all EVs. DIANA miR path software was therefore interrogated to identify the most relevant pathways which, along with Wnt-β-catenin, may contribute to the overlapping of the anti-angiogenic effects detected in antago-miR-24-3p-, pre-miR214-3p-, and anti-IL-3R-EV-treated animals (Fig. 6b). In particular, pathways correlating with downregulated miRs shared by anti-IL-3R-EVs, pre-miR-214-3p-EVs and antago-miR-24-3p-EVs, or by anti-IL-3R-EVs and pre-miR-214-3p-EVs, or anti-IL-3R-EVs and antago-miR-24-3p-EVs were analyzed. As shown in Fig. 8a and resumed in Fig. 8b, miR-222-3p, miR-16-5p, miR-484, miR-17-5p, miR-106a-5p, miR-365b-5p, miR-196b-5p, miR-19b-3p, miR-197-3p, and miR-193b-3p significantly correlated with at least 2 of the following pathways: pathway in cancer, adherens junction, p53 signaling pathway, cell cycle and ECM receptor interaction; whereas, miR-518d-5p and miR-191-5p did not. Of note, miR-16-5p, commonly shared by pre-miR-214-3p-EVs, antago-miR-24-3p-EVs, and anti-IL-3R-EVs significantly correlated with 4 of the above pathways. Such significant correlation was also found for miR-17-5p (in pre-miR-214-3p-EVs) and miR-193b-3p (in antago-miR-24-3p-EVs).

## Discussion

The microenvironment is a complex and dynamic tissue in tumor. It contains neoplastic and resident cells, including stroma and vascular cells [43]. A host of soluble factors have been widely found to modulate this environment. We have previously shown that IL-3 can promote EC proliferation and in vivo angiogenesis [44]. Moreover, it has been shown that signals emanated by IL-3, released by tumor infiltrating T-cells, promote in vivo tumor expansion by increasing tumor neovascularization [3, 45]. However, intercellular crosstalk can also occur in the tumor microenvironment via direct cell-to-cell contact or via mediators that have secreted into the extracellular environment [13]. EVs have become recognized as an important part of cellular communication between cancer cells and cells within the tumor microenvironment [14, 20, 46]. It is becoming evident that EV-mediated communication has a major influence on many aspects of tumor growth and progression [13, 14, 19]. Indeed, EVs released from cancer cells, from stroma and vascular cells can promote tumor growth even by inducing tumor angiogenesis [14–16, 18].

TECs diverge from their normal counterpart at the molecular and functional level [12]. In fact, TECs show rapid turnover and express embryonic gene and vessel growth regulating molecules [12]. Moreover, TECs derived from differing tissues, including breast and kidney tumors, produce IL-3 and are dependent on IL-3 for their growth [10, 12]. We provide a proof of concept, in the present study, for the proangiogenic properties of TEC-derived EVs as well. This observation indicates that, as in normal ECs exposed to inflammatory stimuli [22], EVs released by TECs can act locally as paracrine proangiogenic mediators. An anti-IL-3R blocking antibody [36] was therefore used to investigate whether IL-3R blockade can be therapeutically effective in preventing and/or impairing the growth of tumor vessels that have already formed by interfering with TEC-derived EVs. This may well be quite significant as an efficient blood supply is required for cancer cells to grow beyond a critical mass and progress to a malignant phenotype [46, 47]. Indeed, we have proven that anti-IL-3R-EVs, besides preventing the formation of tumor vessels in vivo, are also able to impair the growth of TEC-derived tumor vessels. EVs are known to influence the behavior of other cells by transferring lipids, proteins, mRNAs, transcription factors and miRs [13, 15, 37–39]. This behavior suggests that IL-3R blockade somehow alters EV biological activity by changing their cargo. Genome-wide profiling has revealed that miRs are frequently abnormally expressed in human cancers [48, 49]. Moreover, it is known that miRs are involved in tumorigenesis, metastasis, chemo-resistance and angiogenesis [48–51]. TEC-derived EVs were therefore analyzed for their miR cargo. Indeed, a number of modulated miRs, as compared to naive EVs, were found in anti-IL-3R-EVs. There is an ever increasing amount of evidence to suggest that miRs could work differentially in human tumors. In fact, context-dependent and target-dependent miR function has been reported [52]. Analyses of the pathways associated with anti-IL-3R-EV-miR expression/modulation have led us to the idea that some may potentially play a critical role in our model. Attention was focused on miRs involved in the regulation of the Wnt/β-catenin signaling pathway in order to identify a crucial and less complex network of miRs working in combination. The canonical Wnt/β-catenin pathway can be positively or negatively regulated by miRs at multiple levels [24, 27]. As shown in this study, a number of miRs carried by anti-IL-3R-EVs are able to target the Wnt/β-catenin pathway [24, 27]. Two of these, miR-214-3p, which directly targets β-catenin [40], and miR-24-3p, which targets two members of the “β-catenin interacting complex” (APC and GSK3β) [41] were chosen for the study. The “destruction complex” controls the stability of cytoplasmic β-catenin. When the Wnt pathway is not engaged, CK1 and GSK3β phosphorylate Axin-bound β-catenin to drive β-catenin to the proteasome for disruption. In this study, we demonstrate that APC and GSK3β as well as S33-S37-phosphorylated β-catenin content increased in TECs as the result of anti-IL-3R-EV treatment. Such specific phosphorylated sequence element, at the N-terminal domain of β-catenin, corresponds to the consensus β-TrCP recognition site required for SCF β-TrCP E3 ubiquitin ligase-mediated degradation [53–55]. We herein provided evidences for the formation of a pβ-catenin/β-TrCP complex as the result of anti-IL-3R-EV treatment. Moreover, it is demonstrated that the effects of anti-IL-3R-EVs may be recapitulated by transfecting cells with antago-miR-24-3p-EVs or using EVs that are depleted of miR-24-3p to stimulate TECs. Furthermore, the transfection of TECs with pre-miR-214-3p and the treatment of TECs with EVs that over-express miR-214-3p led to a reduction in β-catenin content. The same effects were observed upon anti-IL-3R-EV treatment. β-catenin, in the nucleus, functions as a transcription factor to activate the expression of cell proliferation, migration and survival genes, such as c-myc and cyclin D1 [29, 32–34]. c-myc was found to be upregulated in naive EV-treated TECs, whereas it was downregulated in TECs stimulated with anti-IL-3R-EVs, antago-miR-24-3p-EVs and pre-miR-214-3p-EVs, which is consistent with what is known on the integrated miR-24-3p and miR-214-3p interaction-network. c-myc is involved in the regulation of proliferation, growth, differentiation and cell survival. It also plays a role in vascular maturation and stabilization, as it is involved in vasculogenesis and angiogenesis [56–58]. c-myc has often been found to be deregulated and/or overexpressed in transformed cells. Our findings suggest that EVs may contribute to c-myc deregulation, even in tumor vessels, via epigenetic mechanisms that target the Wnt/β-catenin pathway.

The growing amount of information available on the Wnt/miRs regulatory network points to the existence of a complex circuit in cancer cells [24, 25, 30]. An analysis of the modulated miRs in anti-IL-3R-EVs uncovered many predictable miRs which post-transcriptionally control the Wnt/β-catenin pathway at different levels, including miR-200a, miR-126, miR-130 and many others [24, 25, 27, 30, 59, 60]. Although we cannot exclude the possibility that EV inducing effects may depend largely on the combination of all the miRs taken together, a comparison of the miR carried by antago-miR-24-3p-EVs, pre-miR-214-3p-EVs, anti-IL-3R-EVs and their functional activity would appear to point to the crucial role played by selective enrichment pathways. As a matter of fact, antago-miR-24-3p-EVs, which were enriched in miR-214-3p, were also much more effective in their anti-angiogenic action than pre-miR-214-3p-EVs. Moreover, a further and deeper comparison among pre-miR-214-3p-EV, antago-miR-24-3p-EV and anti-IL-3R-EV miR content led us to hypothesize that their overlapping anti-angiogenic effects might also depend on the combined action of a pattern of shared miRs (miR-222-3p, miR-16-5p, miR-484 for all EV samples; miR-19b-3p miR-17-5p, miR-196b-5p, miR-365b-5p for pre-miR-214-3p-EVs and anti-IL-3R-EVs; miR-106a-5p, miR-197-3p, miR-193b-3p for antago-miR-24-3p-EVs and anti-IL-3R-EVs) which may be involved in the regulation of a network of genes related to cancer development/progression. Moreover, we found that miR-16-5p, commonly shared by pre-miR-214-3p-EVs, antago-miR-24-3p-EVs, and in anti-IL-3R-EVs significantly correlated with 4 out of the 5 pathways identified by DIANA miR path software. Finally, our results suggest that miR-24-3p may be involved in the reverse epigenetic silencing of miR-214-3p, which moves to EVs from cells. Therefore, besides supporting the notion that silencing or deleting miRs widely modifies the cellular miR network, our results also raise additional concerns about using antago-miRs as therapeutics [61].

The transfer of EV-miR cargo into recipient cells is a potent mechanism that can support cancer cell survival and/or promote angiogenesis [14, 20, 49] We herein provide evidence to show that, just like tumor cells, EVs released from TECs acquire unique miR-cargo features which account for their paracrine mechanism of action. We also identify the Wnt/β-catenin pathway as a crucial regulator of TEC-derived EV proangiogenic action. Moreover, our data indicate that the IL-3R blockade may be a pharmacological option that interferes with TEC-derived EV proangiogenic action. Finally, there is clear interest in combining miR therapeutics, delivered by small-carriers with conventional cytotoxic agents, and molecularly targeted drugs [61]. The IL-3R blockade may have the advantage of combining standard-of-care treatments with an already established and efficient EV-miR package which can enhance antitumor effects.

## Materials and methods

### Reagents

A detailed list of reagents and antibodies is reported in Supplementary Table 5.

### Cell culture

Human breast-derived TECs were isolated and grown as previously described [12, 62, 63]. In selected experiments, starved TECs were cultured (24 h) in the presence of EVs, anti-IL-3R-EVs or EVs that had either been depleted or enriched with miRs (7 × 10^3^ EVs/target cell) [22]. Untreated TECs served as controls. A Limulus amebocyte assay (concentration < 0.1 ng/ml) (Charles River Laboratories, Inc., Wilmington, MA, USA) was used to test possible contamination. Details are in Supplementary Information.

All experiments were performed in accordance with European Guidelines and policies and approved by the Ethical Committee of the University of Turin.

### Isolation and quantification of TEC-derived EVs

EVs were collected from EBM-cultured TECs which were starved and FCS deprived for 24 h. To obtain IL-3R blockade an anti-IL-3Rα neutralizing antibody (R&D Systems, Minneapolis, MN, USA) was used. Trypan blue was used to evaluate cell viability at the end of each experiment (95 ± 3% viable cells/experiment) [17, 37]. After a first centrifugation, cell-free supernatants were submitted to differential ultracentrifugation. EV protein content was quantified as previously described [17]. A Limulus amebocyte assay (concentration < 0.1 ng/ml) was also used. EV size distribution analyses were performed using a NanoSight LM10 (NanoSight Ltd, Minton Park, UK) [64]. Results were displayed as previously described [64]. EVs were also collected for loss- or gain-of-function experiments, as described below. Details are in Supplementary Information.

### EV internalization by TECs

Confocal microscopy (LSM5-PASCAL; Zeiss, Oberkochen, Germany) was used to evaluate EV internalization. Red fluorescent PKH26 dye was used to label a pool of EV particles. Such labeled EVs were processed as previously described [22]. On the basis of our preliminary results 7 × 10^3^ EV/target/cell concentration, were used. Details are in Supplementary Information.

### Cell proliferation assay

The proliferative activity of TECs, treated as indicated, was assayed as previously described (number ± SD of cells per field, 10× magnification) [12, 17, 65].

### Western blot and co-immunoprecipitation analysis

TECs and TEC-derived EVs were lysed and protein concentrations obtained as previously described [22, 66, 67]. 50 μg protein for cells and 10 μg for EVs were processed as previously described [65]. In selected experiments protein-A-Sepharose beads were used for co-immunoprecipitation experiments [67]. Protein levels were normalized to β-actin [68] or Histone-3 (H3). To evaluate β-catenin activity, cytoplasmic and nuclear extracts [67] from TECs, either treated or left untreated as indicated, were prepared as originally described [69]. The presence of the anti-IL-3R antibody bound to TEC-derived EVs or retained in the EV-depleted CM, was also investigated by using an anti-mouse IgG. To this end EVs recovered from 100k g ultracentrifugation were directly subjected to Western blot analysis, while the supernatants were additionally ultracentrifuged for 24 h at 100 kg to obtain EV-depleted CM. These CM were 50× concentrated and analyzed by western blot.

### RNA isolation and quantitative real-time PCR

Total RNA was isolated from TECs, either treated or left untreated as indicated [17]. RNA, from cells and EVs, was then retro-transcribed using TaqMan microRNA RT kits, specific for miR-214-3p and miR-24-3p, and subjected to quantitative real-time PCR (qRT–PCR) [64]. The small nuclear RNA, RNU6B was used to normalize miR expression. In order to collect EVs, depleted of miR-24-3p or enriched in miR-214-3p, loss-of-function and gain-of-function experiments were performed in TECs, as previously described [22, 64]. Details are in Supplementary Information.

### miR screening

TEC-derived EVs, anti-IL-3R-EVs, antago-miR-24-3p-EVs and pre-miR-214-3p-EVs (triplicate of 3 different preparations per sample) were analyzed for their miR content by qRT–PCR using the Applied Biosystems TaqManH MicroRNA Assay Human Panel Early Access kit (Life Technologies). The expression profile of 375 human mature miRs was examined via reverse transcription (Megaplex RT Pools; Life Technologies) using an Applied Biosystems 7900 H qRT–PCR instrument, as previously described [70]. Raw Ct values obtained by qRT–PCR were calculated using the SDS software version 2.3. A comparison of miR expression was conducted using the Expression Suite software (Life Technologies). Fold change in miR expression, across all samples, was calculated as 2^−ΔΔCt^ using basal EVs as control and by normalizing the data using global normalization [71]. The expression of miRs of interest in the analysis was confirmed using the TaqMan microR specific assay kit (Applied Biosystems, Foster City, CA, USA), as described below. Details are in Supplementary Information.

### Tube-like structure formation (in vitro angiogenesis assay)

Growth factor-reduced Matrigel matrix was used to evaluate tube-like structure formation in TECs that were either treated with EVs, anti-IL-3R-EVs (7 × 10^3^ EV/target cell, which was found effective in preliminary experiments performed with different EV numbers ranging from 5 × 10^3^ to 1 × 10^4^) or left untreated [72]. The number of tube-like structures formed was evaluated as detailed in Supplementary Information.

### Study approval

Animal studies were conducted in accordance with the Italian National Institute of Health Guide for the Care and Use of Laboratory Animals (protocol no: 944/2015-PR). Mice were housed according to the Federation of European Laboratory Animal Science Association Guidelines and the Ethical Committee of the University of Turin. All experiments were performed in accordance with relevant guidelines and regulations.

### In vivo angiogenesis assay

To evaluate the in vivo angiogenic potential of TEC-derived EVs, 8 weeks old male SCID mice (Charles River Laboratories Italia Srl, Calco, Italy) (randomized in three groups; eight mice/each group) were injected s.c. into the flank with a growth factor-reduced Matrigel matrix containing 1 × 10^6^ TECs and 1 × 10^5^/cell of different EVs or saline. The number of EVs was selected from preliminary results obtained by using 0.5 × 10^5^/cell, 1 × 10^5^/cell, and 2 × 10^5^/cell (data not shown). Since no differences were detected between 1 × 10^5^/cell and 2 × 10^5^/cell the lower doses was used. Matrigel plugs were removed and processed on day 7 after injection. In order to evaluate the in vivo ability of EVs to induce vessel regression, TEC-derived EVs or anti-IL-3R-EVs, or alternatively antago-miR-24-3p-EVs or pre-miR-214-3p-EVs alone or in combination (1 × 10^5^/injected cell), re-suspended in 20 μl of saline, were directly injected into the Matrigel plugs of SCID mice (randomized in 3 or 6 groups; 8 mice/each group) on days 3 and 7 after TEC implantation (1 × 10^6^ cells). An equal volume of saline was used in control mice. Matrigel plugs were removed and processed after 10 days [12, 72]. Details are in Supplementary Information.

### Bioinformatic analyses

Ingenuity Pathway Analysis (IPA) Software (Ingenuity Systems, Qiagen, Redwood City, CA) was used to generate the interaction-network that correlated the miRs of interest and mRNAs.

The DIANA-miRPath v3.0 database was interrogated by setting up KEGG annotations to select miR targeting pathways, according to their enrichment in p-values. Each miR and interaction dataset were entered and examined individually or together with other significant miRs. The merging algorithm was considered where appropriate [73].

### Statistical analysis

All data are reported as mean ± SD. The D’Agostino–Pearson test was used to test normality. Unpaired student *t*-tests were used to compare two experimental groups, while one-way ANOVA, followed by Tukey’s multiple comparison test, to compare ≥ 3 experimental groups. The differences in the fold induction of protein contents were evaluated by densitometric analyses and reported as relative amount. For the in vitro experiments the minimum sample size to ensure 90% power between controls and experimental groups with a probability level of 0.05, two-tailed hypothesis, was three experiments performed in triplicate. For the in vivo experiments the minimum sample size to detect a 40% difference between controls and experimental groups, with 90% power and a probability level of 0.05 in a two-tailed hypothesis, was *n* = 8 mice/group. *P*-values < 0.05 were considered statistically significant (*p < 0.05, **p < 0.01, ***p < 0.001). Three independent and blinded investigators evaluated the in vivo outcomes. All statistical analyses were carried out using GraphPad Prism version 5.04 (GraphPad Software, Inc., La Jolla, CA, USA).

## Electronic supplementary material

Supplementary Information
