# Supplementary material for: IL-3R-alpha blockade inhibits tumor endothelial cell-derived extracellular vesicle (EV)-mediated vessel formation by targeting the β-catenin pathway
Source: Oncogene. 2017 Dec 14;37(9):1175–91. doi: 10.1038/s41388-017-0034-x (PMC5861089; doi:10.1038/s41388-017-0034-x)
Supplement: Supplementary file 1 — Supplementary Information [file 41388_2017_34_MOESM1_ESM.docx]

**Supplementary Table 1**

**Supplementary Table 1.** Significantly down-regulated miRs (fold change values ≤ -2 ±SD in anti-IL-3R-EVs normalized to EVs) are listed.

**Supplementary Table 2**

**Supplementary Table 2.** Significantly up-regulated miRs (fold change values ≥ 2 ±SD in anti-IL-3R-EVs normalized to EVs) are listed

**Supplementary Table 3**

**Supplementary Table 3.** Fold decrease/increase in miRs expressed by antago-miR-24-3p-EVs normalized to EVs are listed.

**Supplementary Table 4**

**Supplementary Table 4.** Fold increase/decrease in miRs expressed by pre-miR-214-3p-EVs normalized to EVs are listed

**Supplementary Table 5**

| **REAGENTS** | | |
| --- | --- | --- |
| **Description:** | **Purchased from:** | |
| FBS (F6178), SDS (L3771), PIPES (P9291), Triton X-100 (T8787), Nonidet P-40 (74385), NaCl (S3014), NaF (S7920), Na_3_OV_4_ (S6508), Na_4_P_2_O_7_ (P8010), MgCl_2_ (M8266), KCl (P9541), HCl (258148), Na-azide (S2002), Hepes (H3375), Tris (T1503), EDTA (E6758), EGTA (E4378), ethanol (51976), aprotinin (A6279), pepstatin A (P5318), PMSF (P7626), DMSO (D8418), PKH26 dye (MINI26-1KT), leupeptin (L2884), penicillin-streptomycin (P4333), Trypsin (T4799), protein A–Sepharose beads (P3391) | Sigma-Aldrich (St Louis, MO, USA) | |
| EBM Basal Medium (CC-3121) | Lonza Inc (Allendale, NJ, USA) | |
| Protein molecular weight markers (161-0374), Acrylammide (161-0156), polyvinylidene difluoride (PVDF) membranes (162-0115), Bradford reagent (500-0205), ECL (170-5061) | Bio-Rad (Hercules, CA, USA) | |
| Matrigel Basement Membrane Matrix Growth Factor Reduced (356231) | BD Bioscience Pharmingen (Franklin Lakes, NJ, USA) | |
| Lipofectin® Reagent (18292-037), TRIZOL (15596018), RNU6B (001093), hsa-miR-214-3p (002306), hsa-miR-24-3p (000402), has-miR-24 Anti-miR™ miRNA Inhibitor (AM10737), hsa-miR-214 Pre-miR™ miRNA Precursor (PM12124), Opti-MEM (11058021) | Invitrogen^TM^ (Life Technologies Carlsbad, CA, USA; Paisley, UK). | |
| **ANTIBODIES** | | |
| **Description:** | **Purchased from:** | |
| anti-β actin (sc-47778), anti-cyclin D1 (sc-20044), anti-CD81 (sc166029) | S. Cruz Biotechnology (Heidelberg, Germany) | |
| anti-GSK3 β (ab93926), anti-APC (ab40778), anti-phospho S33 + S37 β catenin (ab11350), anti-β catenin (ab16051), anti-c-myc (ab32072), anti-CD63 (ab134045), anti-histone-H3 (ab1791) | Abcam (Cambrige, UK) | |
| Human IL‑3 R alpha Antibody (Mouse) (MAB301) | R&D System Inc. (Minneapolis, MN, USA) | |
| anti-rabbit IgG, HRP linked (4050-05)  anti-mouse IgG, HRP linked (1031-05) | Southern Biotech (Birmingham, Alabama USA) | |
| β-TrCP (D13F10) , Phospho-β-Catenin (Ser33/37) Antibody #2009 | | Cell Signaling (EuroClone**,** Milan, Italy) |

**Isolation and quantification of TEC-derived EVs**

TECs were cultured in EBM, without FBS, for 24h either with or without the anti-IL-3Rα neutralizing antibody in order to collect EVs from supernatants. After being centrifuged at 3000 g for 30 min to remove debris, cell-free supernatants were submitted to differential ultracentrifugation at 10 000 and 100 000g (Beckman Coulter Optima L-90K ultracentrifuge; Beckman Coulter, Fullerton, CA, USA) for 3h at 4°C. EVs were either used fresh or stored (-80°C) after re-suspension in EBM supplied with 1% DMSO^1,2^. Frozen EVs were washed and pelleted by 100k g ultracentrifugation to remove DMSO before experiments. No differences in biological activity between fresh and stored EVs were observed. EV protein content was quantified using the Bradford method (Bio-Rad, Hercules, CA, USA). Possible contamination was tested for using a Limulus amebocyte assay (concentration <0.1 ng/ml) (Charles River Laboratories, Inc., Wilmington, MA, USA). EV size distribution analyses were performed using a NanoSight LM10 (NanoSight Ltd, Minton Park UK). The particles in the samples were illuminated using a laser light source and the scattered light was captured by camera and analyzed using Nanoparticle Tracking Analysis (NTA). NTA automatically tracked and sized particles according to Brownian motion and the diffusion coefficient (Dt)^3^. Results were displayed as a frequency size distribution graph and outputted to a spreadsheet. EVs were also collected from TECs subjected to loss- or gain-of-function experiments, as described below.

**EV internalization by TECs**

The internalization of EVs into TECs was evaluated using confocal microscopy (LSM5-PASCAL; Zeiss, Oberkochen, Germany). A pool of EV particles was labeled with red fluorescent PKH26 dye (2 μl ml−1) for 30 min at 37 °C and EVs were then washed and ultracentrifuged at 100 000 g for 1 h at 4 °C. EV pellets were suspended in DMEM and added, at 7×10^3^ EV/target cell concentration, to TECs for 3h in order to detect internalization^4^. The EV number was selected on the bases of our preliminary experiments performed by using EV number ranging from 5×10^3^ to 1×10^4^.

**Western blot and co-immunoprecipitation analysis**

TECs and TEC-derived EVs were lysed and protein concentrations obtained as previously described^4^. Nuclear extracts were prepared as previously described^5^. 50 μg protein for cells and 10 μg for EVs were subjected to SDS-PAGE, transferred into nitrocellulose membranes and processed as previously described^5^. In selected experiments protein-A-Sepharose beads were used for co-immunoprecipitation experiments^5^. Densitometric analysis was used to calculate the differences in the fold induction of protein levels which were normalized to β-actin^6^ or Histone-3 (H3). Values are reported as relative amounts^5^. To evaluate β-catenin activity, cytoplasmic and nuclear extracts from TECs, either treated or left untreated as indicated, were prepared as originally described by Sadowski *et al*^7^_._ The presence of the anti-IL-3R antibody bound to TEC-derived EVs or retained in the EV-depleted CM, was also investigated by using an anti-mouse IgG. To this end EVs recovered from 100k g ultracentrifugation were directly subjected to western blot analysis, while the supernatants were additionally ultracentrifuged for 24 h at 100k g to obtain EV-depleted CM. These CM were 50X concentrated and analyzed by western blot.

**RNA isolation and quantitative real-time PCR (qRT-PCR)**

Total RNA was isolated from TECs, either treated or left untreated as indicated, using the TRIzol reagent (Invitrogen) and from indicated EVs using the mirVana RNA Isolation Kit (Ambion), according to manufacturer's instructions^2^_._ RNA was quantified spectrophotometrically (Nanodrop ND-1000, Wilmington, DE, USA) because intact 18S and 28S rRNAs were difficult to detect in the EVs. RNA, from cells and EVs, was then retrotranscribed using TaqMan microRNA RT kits, specific for miR-214-3p and miR-24-3p, and subjected to qRT-PCR using a TaqMan microRNA assay kit and the ABI PRISM 7700 sequence detection system (Applied Biosystems, Foster City, CA, USA). miR expression was normalized to the small nuclear RNA, RNU6B. In order to collect EVs, depleted of miR-24-3p or enriched in miR-214-3p, loss- and gain-of-function experiments were performed in TECs transfected for 48h with the antago-miR negative control, the antago-miR-24-3p, the pre-miR negative control or pre-miR-214-3p oligonucleotides (Applied Biosystem), according to manufacturer’s instructions^3,4^.

**miR Screening**

TEC-derived EVs, anti-IL-3R-EVs, antago-miR-24-3p-EVs and pre-miR-214-3p-EVs (triplicate of 3 different preparations per sample) were analyzed for their miR content by quantitative real time (qRT) PCR using the Applied Biosystems TaqManH MicroRNA Assay Human Panel Early Access kit (Life Technologies). The expression profile of 375 human mature miRs was examined via sequential steps of reverse transcription (Megaplex RT Pools; Life Technologies) using an Applied Biosystems 7900H qRT-PCR instrument, as previously described^8^. Briefly, single stranded cDNA was generated from a total RNA sample (80 ng), obtained as above, by reverse transcription using a mixture of looped primers (Megaplex RT kit, Life Technologies) according to manufacturer’s instructions. The pre-amplification reaction for each sample was performed using a TaqMan® PreAmp Master Mix 2X (Life Technologies) mixed with specific Megaplex™ PreAmp Primers (10X) (Life Technologies). Pre-amplified products were then diluted, loaded into the TaqMan MicroRNA Array and qRT-PCR experiments were performed. Raw Ct values were calculated using the SDS software version 2.3. A comparison of miR expression was conducted using the Expression Suite software (Life Technologies). Fold change in miR expression, across all samples, was calculated as 2^-ΔΔCt^ using basal EVs as control and by normalizing the data using global normalization^9^. The expression of miRs of interest in the analysis was confirmed using the TaqMan microR specific assay kit (Applied Biosystems, Foster City, CA, USA), as described below.

**Tube-like structure formation (*in-vitro* angiogenesis assay)**

24-well-plates were coated with growth factor-reduced Matrigel matrix to analyze tube-like structure formation in TECs that were either treated with EVs, anti-IL-3R-EVs (7×10^3^ EV/target cell which was found effective in preliminary experiments performed with different EV numbers ranging from 5 ×  10^3^ to 1 ×  10^4^ ) or left untreated. After 24h, the number of tube-like structures formed was evaluated by three different operators (each experiment was performed in triplicate), counting 10 fields at 10X magnification, using a phase-contrast microscope (Leica DMIL, Wetzlar, Germany).

***In-vivo* angiogenesis assay**

In order to evaluate the *in-vivo* angiogenic potential of TEC-derived EVs, SCID mice (Charles River Laboratories Italia Srl, Calco, LC, Italy) (four mice for each experimental group) were injected s.c. into the flank with a growth factor-reduced Matrigel matrix containing 1×10^6^ TECs and 1×10^5^/cell of different EVs or saline. The number of EVs was selected from preliminary results obtained by using 0.5×10^5^/cell, 1×10^5^/cell, and 2×10^5^/cell (data not shown). Since no differences were detected between 1×10^5^/cell and 2×10^5^/cell the lower doses was used (data not shown). Matrigel plugs were removed and processed on day 7 after injection. In order to evaluate the *in-vivo* ability of EVs to induce vessel regression, either TEC-derived EVs or anti-IL-3R-EVs (1×10^5^/injected cell), re-suspended in 20 µl of saline, were directly injected into the Matrigel plugs of SCID mice (four mice for each experimental group) on days 3 and 7 after TEC implantation (1×10^6^ cells). An equal volume of saline was used in control mice. Matrigel plugs were removed and processed after 10 days. Recovered Matrigel plugs were ﬁxed in 10% buffered formalin and embedded in parafﬁn for histological analyses. 5 μm thick paraffin Matrigel sections were routinely stained with Masson’s Trichrome blue (Dako), according to manufacturer’s instructions. The quantification of neoformed vessels was expressed as the number±SD of vessels per sample (20X magnification)^10^. Only vessels possessing a patent lumen that contained red blood cells were considered for the study^11^.

**REFERENCES**

1. Deregibus MC, Cantaluppi V, Calogero R, Lo Iacono M, Tetta C, Biancone L, *et al*. Endothelial progenitor cell derived microvesicles activate an angiogenic program in endothelial cells by a horizontal transfer of mRNA. *Blood* 2007; **110**: 2440–2448.
2. Togliatto G, Dentelli P, Gili M, Gallo S, Deregibus C, Biglieri E, *et al*. Obesity reduces the pro-angio-genic potential of adipose tissue stem cell-derived extracellular vesicles (EVs) by impairing miR-126 content: impact on clinical applications. *Int J Obes* 2016; **40**: 102–111.
3. Gallo S, Gili M, Lombardo G, Rossetti A, Rosso A, Dentelli P et al. Stem Cell-Derived, microRNA-Carrying Extracellular Vesicles: A Novel Approach to Interfering with Mesangial Cell Collagen Production in a Hyperglycaemic Setting. *PLoS One* 2016; **11**: e0162417.
4. Lombardo G, Dentelli P, Togliatto G, Rosso A, Gili M, Gallo S, *et al*. Activated Stat5 trafficking Via Endothelial Cell-derived Extracellular Vesicles Controls IL-3 Pro-angiogenic Paracrine Action. *Sci Rep* 2016; **6:** 25689**.**
5. Trombetta A, Togliatto G, Rosso A, Dentelli P, Olgasi C, Cotogni P et al. Increase of palmitic acid concentration impairs endothelial progenitor cell and bone marrow-derived progenitor cell bioavailability: role of the STAT5/PPARγ transcriptional complex. *Diabetes* 2013; **62**: 1245-1257.
6. Bruno S, Tapparo M, Collino F, Chiabotto G, Deregibus MC, Soares Lindoso R. Renal Regenerative Potential of Different Extracellular Vesicle Populations Derived from Bone Marrow Mesenchymal Stromal Cells. *Tissue Eng Part A* 2017
7. Sadowski HB, Shuai K, Darnell JE Jr, Gilman MZ. A common nuclear signal transduction pathway activated by growth factor and cytokine receptors. *Science* 1993; **261**: 1739–1744.
8. Collino F, Pomatto M, Bruno S, Lindoso RS, Tapparo M, Sicheng W, et al. Exosome and Microvesicle-Enriched Fractions Isolated from Mesenchymal Stem Cells by Gradient Separation Showed Different Molecular Signatures and Functions on Renal Tubular Epithelial Cells. *Stem Cell Rev* 2017; **13**: 226-243.
9. Mestdagh P, Van Vlierberghe P, De Weer A, Muth D, Westermann F, Speleman F, Vandesompele J. A novel and universal method for microRNA RT-qPCR data normalization. *Genome Biol* 2009; **10**: R64.
10. Dentelli P, Rosso A, Olgasi C, Camussi G, Brizzi MF. IL-3 is a novel target to interfere with tumor vasculature. *Oncogene* 2011; **30**: 4930–4940.
11. Cavallari C, Ranghino A, Tapparo M, Cedrino M, Figliolini F, Grange C, *et al*. Serum-derived extracellular vesicles (EVs) impact on vascular remodeling and prevent muscle damage in acute hind limb ischemia. *Sci Rep* 2017; **7**: 8180.

**Supplementary Figure 1**

**
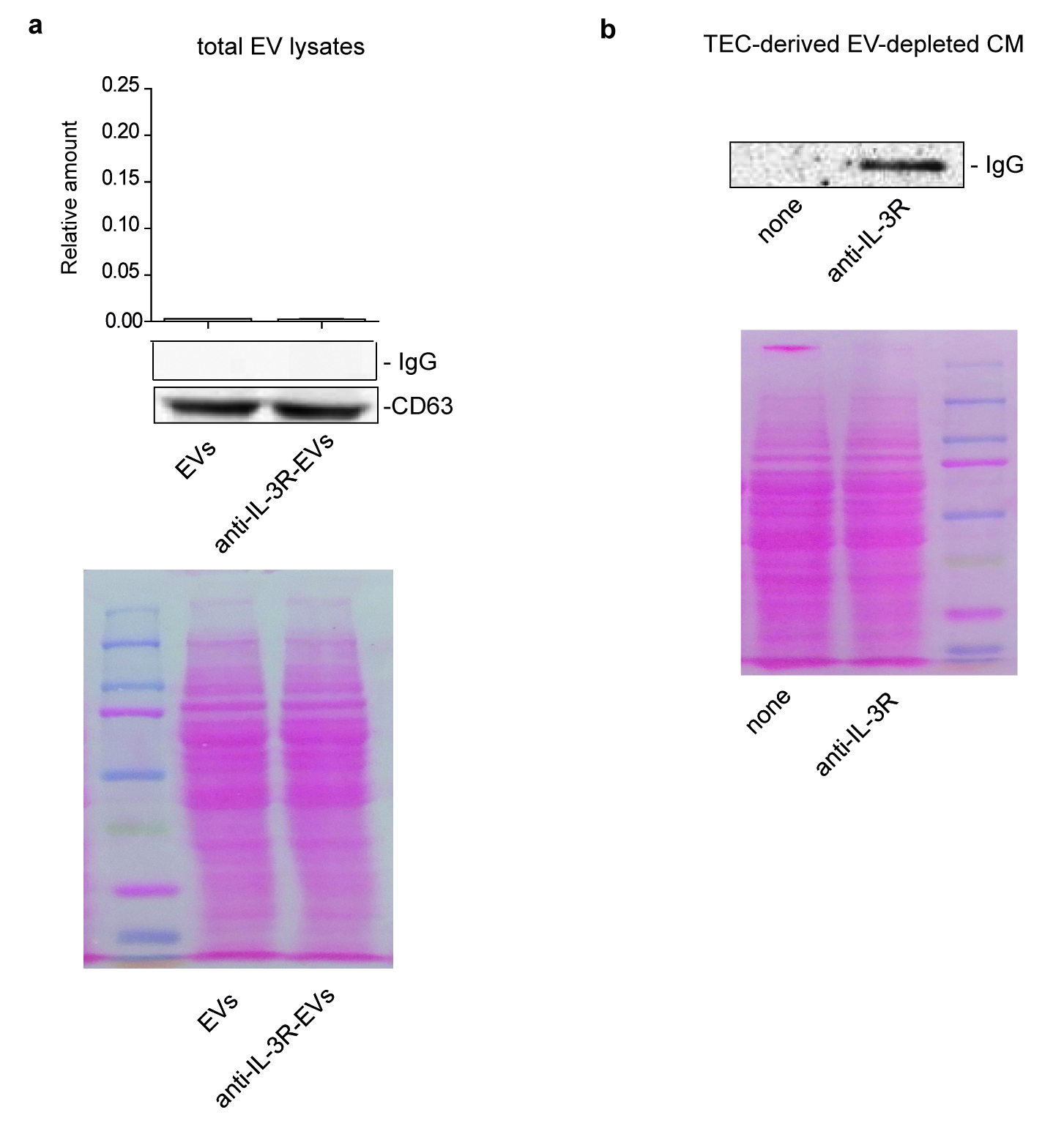
**

**Supplementary Figure 1. (a)** EVs collected from untreated or anti-IL-3R-treated TECs were lysed and analyzed by western blot using an anti-mouse IgG antibody **(b)** The EV-derived CM obtained as described Materials and Methods were subjected to SDS-PAGE and analyzed as above described. Ponceau-stained membranes were used to demonstrate loaded proteins from each sample (EV-lysates and EV-depleted CM) (lower panels).

**Supplementary Figure 2**





**Supplementary Figure 2. (a)** Cell extracts from untreated or anti-IL-3R-treated TECs were analyzed for β-catenin content, normalized to β-actin (n=3) **(b)** TECs treated as above were lysed and analyzed to evaluate GSK 3β, APC and pβ-catenin content, normalized to β-actin (n=3). **(c)** Total EV lysates were subjected to SDS-PAGE to evaluate GSK 3β and APC content, normalized to β-actin (n=3). Total cell lysate from untreated TECs was used as positive control (C).

**Supplementary Figure 3**


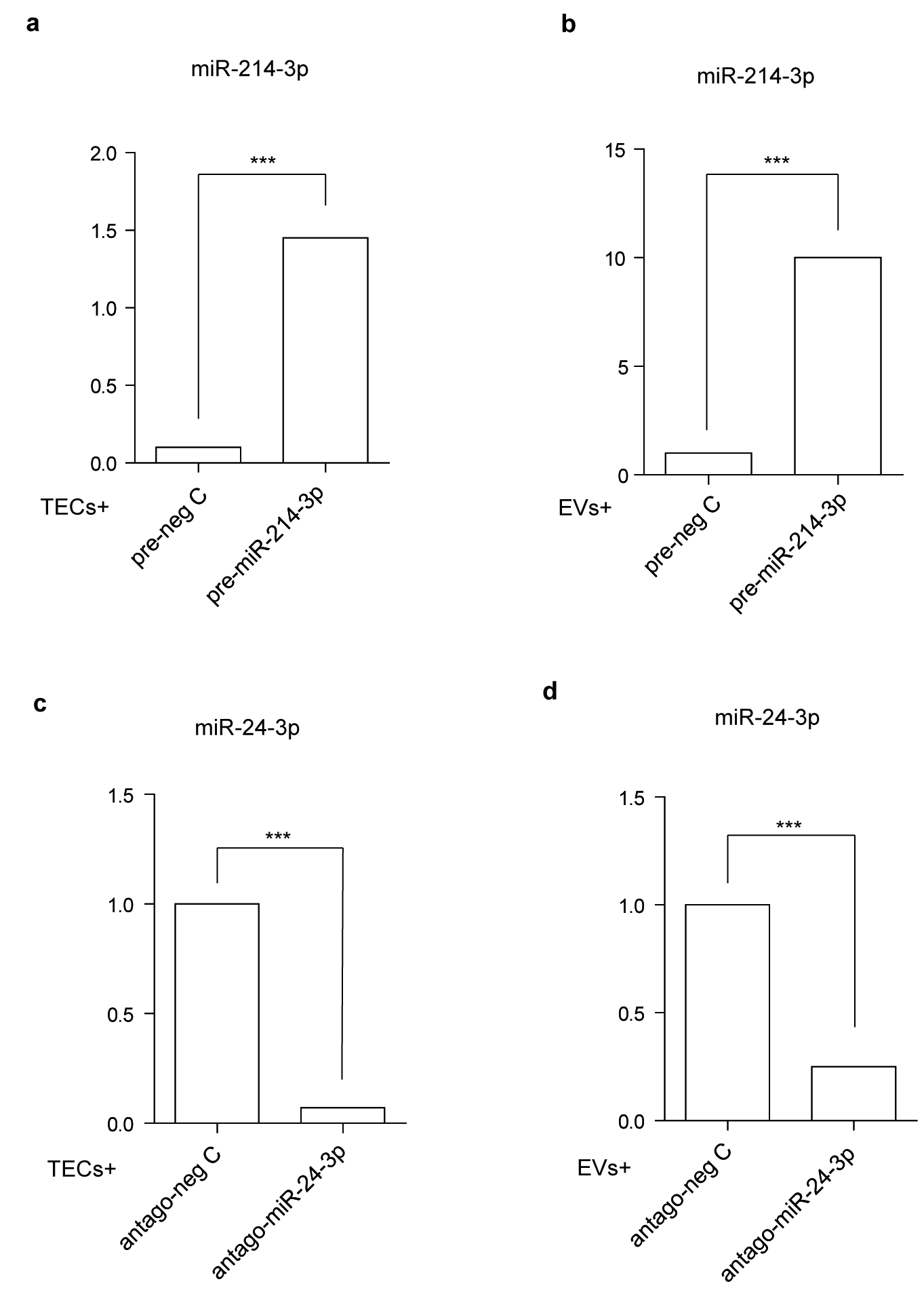


**Supplementary Figure 3. (a-b)** Gain-of-function experiments were performed on TECs after 48h incubation with pre-miR-214-3p oligonucleotides. Pre-miR negative control (pre-neg c) oligonucleotides were used as control to evaluate the expression in both cells and EVs. miR-214-3p expression was evaluated by qRT-PCR either in transfected TECs (**a**) or in TEC-derived EVs (pre-miR-214-3p-EVs) (b). Data are normalized to RNU6B (n=5) (****p<0*.*001*, pre-miR-214-3p vs pre-miR-neg c in (**a**) and (**b**)). **(c-d)** Loss-of-function experiments were performed on TECs after 48h incubation with antago-miR-24-3p. Antago-miR negative control (antago-neg c) was used as control. miR-24-3p expression was evaluated by qRT-PCR either in transfected TECs (**c**) or in TEC-derived EVs (antago-miR-24-3p-EVs) (**d**). Data are normalized to RNU6B (n=5) (****p<0*.*001*, antago-miR-24-3p vs antago-neg c in (**c**) and (**d**)).
